# Squalene epoxidase promotes the chemoresistance of colorectal cancer via (S)-2,3-epoxysqualene-activated NF-κB

**DOI:** 10.1186/s12964-024-01649-z

**Published:** 2024-05-18

**Authors:** Qi Liu, Yajuan Zhang, Huimin Li, Hong Gao, Yijie Zhou, Dakui Luo, Zezhi Shan, Yufei Yang, Junyong Weng, Qingguo Li, Weiwei Yang, Xinxiang Li

**Affiliations:** 1 Department of Colorectal Surgery, Fudan University Shanghai Cancer Center, Shanghai, China; 2 Department of Oncology, Shanghai Medical College, Fudan University, Shanghai, China; 3 Shanghai Institute of Thoracic Oncology, Shanghai Chest Hospital, Shanghai Jiao Tong University School of Medicine, Shanghai, 200030 China; 4 Key Laboratory of Systems Health Science of Zhejiang Province, School of Life Science, Hangzhou Institute for Advanced Study, University of Chinese Academy of Sciences, Hangzhou, China; 5 State Key Laboratory of Cell Biology, Shanghai Key Laboratory of Molecular Andrology, Shanghai Institute of Biochemistry and Cell Biology, Center for Excellence in Molecular Cell Science, Chinese Academy of Sciences, University of Chinese Academy of Sciences, Shanghai, China

**Keywords:** Squalene epoxidase, Chemoretherapy, Colorectal cancer, (S)-2,3-epoxysqualene, NF-κB pathway

## Abstract

**Background:**

While de novo cholesterol biosynthesis plays a crucial role in chemotherapy resistance of colorectal cancer (CRC), the underlying molecular mechanism remains poorly understood.

**Methods:**

We conducted cell proliferation assays on CRC cells with or without depletion of squalene epoxidase (SQLE), with or without 5-fluorouracil (5-FU) treatment. Additionally, a xenograft mouse model was utilized to explore the impact of SQLE on the chemosensitivity of CRC to 5-FU. RNA-sequencing analysis and immunoblotting analysis were performed to clarify the mechanism. We further explore the effect of SQLE depletion on the ubiquitin of NF-κB inhibitor alpha (IκBα) and (S)-2,3-epoxysqualene on the binding of IκBα to beta-transducin repeat containing E3 ubiquitin protein ligase (BTRC) by using immunoprecipitation assay. In addition, a cohort of 272 CRC patients were selected for our clinical analyses.

**Results:**

Mechanistically, (S)-2,3-epoxysqualene promotes IκBα degradation and subsequent NF-κB activation by enhancing the interaction between BTRC and IκBα. Activated NF-κB upregulates the expression of baculoviral IAP repeat containing 3 (BIRC3), sustains tumor cell survival after 5-FU treatment and promotes 5-FU resistance of CRC in vivo. Notably, the treatment of terbinafine, an inhibitor of SQLE commonly used as antifungal drug in clinic, enhances the sensitivity of CRC to 5-FU in vivo. Additionally, the expression of SQLE is associated with the prognosis of human CRC patients with 5-FU-based chemotherapy.

**Conclusions:**

Thus, our finding not only demonstrates a new role of SQLE in chemoresistance of CRC, but also reveals a novel mechanism of (S)-2,3-epoxysqualene-dependent NF-κB activation, implicating the combined potential of terbinafine for 5-FU-based CRC treatment.

**Supplementary Information:**

The online version contains supplementary material available at 10.1186/s12964-024-01649-z.

## Background

Colorectal cancer (CRC) is one of the most common malignant tumors in the world [1]. It ranks third in terms of overall incidence and second in terms of mortality among all cancers. In addition to surgical treatment, chemotherapy plays a significant role in CRC treatment. 5-fluorouracil (5-FU), an antimetabolite, is a fundamental chemotherapy drug for CRC. By inhibiting thymidylate synthase (TS), 5-FU hinders DNA synthesis and exerts its anti-tumor effects [2, 3]. 5-FU-based adjuvant chemotherapy is recommended for all eligible patients with stage III colon cancer after radical resection, as well as high-risk stage II colon cancer patients (poorly differentiated, T4 tumors, tumor perforation and less than 12 lymph nodes retrieved) [4]. However, the efficacy of chemotherapy in CRC remains suboptimal, and most CRC patients ultimately develop drug resistance [5]. In addition, more than 90% of patients diagnosed with metastatic CRC would suffer chemotherapy failure due to drug resistance, and their 5-year survival rate is only 14% [5, 6]. Therefore, elucidating the mechanism of resistance of CRC to 5-FU is of great significance in designing successful chemotherapy regimens and improving the prognosis of patients with CRC.

Cholesterol metabolism plays an indispensable role in mammalian cells, and its dysregulation is closely related to a variety of diseases, including CRC. The lipidic milieu could regulate the activity of essential transmembrane proteins, leading to the resistance to antineoplastic drugs. Additionally, certain metabolites of cholesterol metabolism, such as farnesyl pyrophosphate, mevalonic acid, and geranylgeranyl pyrophosphate, have been implicated in cancer signaling [7–9]. Moreover, inhibition of specific enzymes involved in cholesterol metabolism, including lanosterol synthase (LSS), farnesyl diphosphate synthase (FDPS), and squalene epoxidase (SQLE), has been shown to suppress cancer metastasis [10–15]. Researchers also found that the T lymphocytes were regulated by mevalonate metabolism of lipid biosynthesis [16]. However, the relationship between cholesterol metabolism and chemotherapy has not been extensively studied, with previous studies only focusing on cholesterol itself, neglecting the intermediate products of cholesterol metabolism [17, 18]. The key enzymes in cholesterol synthesis include 3-hydroxy-3-methylglutaryl-coenzyme A reductase (HMGCR) and SQLE, and previous studies suggest that the inhibition of HMGCR could enhance the efficacy of cancer treatments [19].

SQLE, as the second key enzyme in cholesterol biosynthesis, catalyzes the initial oxygenation step [20]. Squalene is converted by SQLE to (S)-2,3-epoxysqualene during cholesterol synthesis, but limited information is currently available regarding the significance of (S)-2,3-epoxysqualene in cancer research [21, 22]. SQLE is overexpressed in several cancers and the high expression of SQLE is closely related to the poor prognosis of cancer patients, including CRC [20, 23]. It is reported that the poor survival of CRC patients with high SQLE expression is due to its promotion of tumor cell proliferation. However, previous studies have not explored the effect of SQLE on CRC treatment efficacy [24]. In this study, we investigate the role and mechanism of SQLE in the resistance of CRC to 5-FU. For the first time, our research has uncovered and demonstrated the inhibitory role of SQLE in CRC sensitivity to 5-FU, thus underscoring SQLE as a promising target for CRC treatment. Through further exploration of the underlying mechanism, we have discovered that the direct product of SQLE-catalyzed squalene, namely (S)-2,3-epoxysqualene, promotes the binding of NF-κB inhibitor alpha (IκBα) to beta-transducin repeat containing E3 ubiquitin protein ligase (BTRC), consequently promoting the upregulation of ubiquitin levels and subsequent degradation of IκBα. This activation of the NF-κB pathway ultimately attenuates the responsiveness of CRC to 5-FU. Beyond presenting a novel therapeutic avenue for combating colorectal cancer, our investigation has notably established the initial connection between a metabolic intermediate of cholesterol metabolism and the NF-κB pathway.

## Methods

### Cell culture

RKO and HEK293T cells were purchased from the Type Culture Collection of the Chinese Academy of Sciences. WiDr cells were purchased from Nanjing COBIOER biosciences Co., Ltd. These cells were cultured with dulbecco's modified eagle medium (DMEM), 10% fetal bovine serum (FBS) and streptomycin penicillin. Cell lines used in the experiments were keep at 37 °C in an incubator with 95% air and 5% CO_2_ supply.

### DNA constructs

Polymerase chain reaction (PCR)-amplified human *SQLE*, *Baculoviral IAP repeat containing 3 (BIRC3)* and *NF-kappa-B inhibitor alpha (NFKBIA)* were cloned into pCDH-Flag and pcDH-HA. The mutations were made using the Quick-Change site-directed mutagenesis kit (Stratagene, La Jolla, CA). The GV248 shNT was generated with the control 5’-TTCTCCGAACGTGTCACGT-3’ oligonucleotide and GV248 shSQLE was generated with 5’-gcTCAGGCTCTTTATGAATTA-3’ oligonucleotide. GV248 SQLE shRNA-2 was generated with 5’- gcAGAGCCCAATGCAAAGTTT -3’ oligonucleotide.

### Immunoprecipitation and immunoblotting analysis

Extraction of proteins with a modified buffer from cultured cells was followed by immunoprecipitation and immunoblotting with corresponding antibodies, as described previously [25].

### Cell proliferation assay

2500 cells per well were plated in 96-well plate and cultured in DMEM with 10% FBS for 3 days. 5-FU was added in each well 24 h after cells were plated with indicated concentrations. Cell proliferation is quantified by Sulforhodamine B (SRB) assay. Briefly, cells were fixed with trichloroacetic acid and stained with SRB solution, followed by rinsing the plates four times with 1% acetic acid and solubilizing the protein-bound dye with 10 mM Tris base solution. The absorbance was measure at 560 nm. Data were shown with the means ± SD of 3 independent experiments.

### Quantitative real-time reverse transcription PCR

TRIzol (Invitrogen, Thermo Fisher Scientific) was used to extract the total RNA, and total RNA was then reverse-transcribed into cDNA using a ReverTra Ace qPCR RT Master Mix Kit (Toyobo) with oligonucleotide (dT) and random primers. Quantitative realtime PCR analysis was performed under the following conditions: 5 min at 95℃ followed by 40 cycles at 95℃ for 30 s, 55 ℃ for 40 s, and 72℃ for 1 min using a Bio-Rad PCR Thermal Cyclers. Then real-time PCR was performed on an Applied Biosystems 7500 using SYBR Green PCR Master mix (Thermo Fisher Scientific). Primer sequences used for the amplification of human *SQLE* were 5′- GGCATTGCCACTTTCACCTAT -3′ (forward) and 5′- GGCCTGAGAGAATATCCGAGAAG -3′ (reverse). Primer sequences used for the amplification of human *BIRC3* were 5′- AAGCTACCTCTCAGCCTACTTT -3′ (forward) and 5′- CCACTGTTTTCTGTACCCGGA -3′ (reverse).

### Subcutaneous injection

In brief, 6-week-old randomized female athymic nude mice were subcutaneously injected with 1 × 10^6^ RKO cells. Five mice were included in each experiment. 23 days after injection, mice were euthanized, and tumors were dissected for the measure of tumor weight. All mice were housed in a specific pathogen-free environment at the Shanghai Institute of Biochemistry and Cell Biology.

### Immunohistochemical analysis

The tissue sections from paraffin-embedded human CRC specimens were stained with antibodies as indicated. We quantitatively scored the tissue sections according to the percentage of positive cells and staining intensity. We scored the staining intensity as follows: 0 (negative), 1 (weak), 2 (moderate) and 3 (strong). The immunohistochemical score (H score) = the percentage of strongly stained area × 3 + the percentage of moderately stained area × 2 + the percentage of weakly stained area × 1, giving a range of 0 to 300.

### Selection of CRC patients

Formalin-fixed paraffin-embedded (FFPE) specimens used in this study were obtained from 272 patients who underwent radical resection of CRC in FUSCC between January 2007 and December 2009. Inclusion criteria for the patient cohort included confirmed colorectal cancer diagnosis and the treatment with 5-FU-based chemotherapy. Patients with unknown tumor grade, unknown venous invasion status or unknown carcinoembryonic antigen (CEA) level were excluded from further Cox proportional hazards analysis. CRC tissue was fabricated into tissue microarrays. The clinicopathological features selected for analysis included TNM stage, age, gender, primary tumor location, preoperative serum CEA level, tumor grade, vascular invasion and the number of lymph nodes examined. These patients received long-term active follow-up and their oncological outcomes were also documented after surgery.

### Statistical analysis

The end points used for clinical analysis in the present study were overall survival (OS) and disease-free survival (DFS). We used chi-square test to analyze the categorical variables. Kaplan–Meier survival analysis was used to analyze the OS and DFS of different groups, and log-rank tests were used to compare the survival differences. We also established Cox proportional hazard models and used Hazard ratio (HR) and 95% confidence interval (CI) to analyze different prognostic factors of CRC patients. The two-tailed t test (normal distribution) or Mann–Whitney test (nonnormal distribution, not paired) or Wilcoxon signed-rank test (nonnormal distribution, paired) was used to analyze the data between the experimental group and the control group. The results were expressed by mean ± standard deviation (SD), and *p* < 0.05 was considered to be statistically significant, *P* < 0.01 was considered to be extremely significant (*p < 0.05, **p < 0.01).

## Results

### SQLE expression correlates with the response of CRC patients to 5-FU-based chemotherapy

Through the Cancer Genome Atlas (TCGA), we obtained the mRNA expression data of SQLE in tumor tissues and peritumoral tissues from colorectal cancer (CRC) patients, and found that the mRNA levels of SQLE were significantly up-regulated in tumor tissues compared with peritumoral tissues (*P* < 0.05, Fig. S1A). We next selected the patients with paired expression information of tumor tissues and adjacent peritumoral tissues, which also showed that tumor tissues had higher mRNA levels of SQLE than paired peritumoral tissues (*P* < 0.01, Fig. S1B). Furthermore, we performed immunohistochemistry (IHC) staining using the CRC tissue microarray from Fudan University Shanghai Cancer Center (FUSCC) after verifying the specificity of anti-SQLE antibody (Fig. S1C). We found that tumor tissues had much higher protein levels of SQLE than peritumoral tissues from CRC patients (*P* < 0.01, Fig. S1D). Similarly, tumor tissues had much higher protein levels of SQLE than paired peritumoral tissues from CRC patients (*P* < 0.01, Fig. S1E). These results confirm the association of SQLE with the malignancy of CRC.

**Fig. 1:**
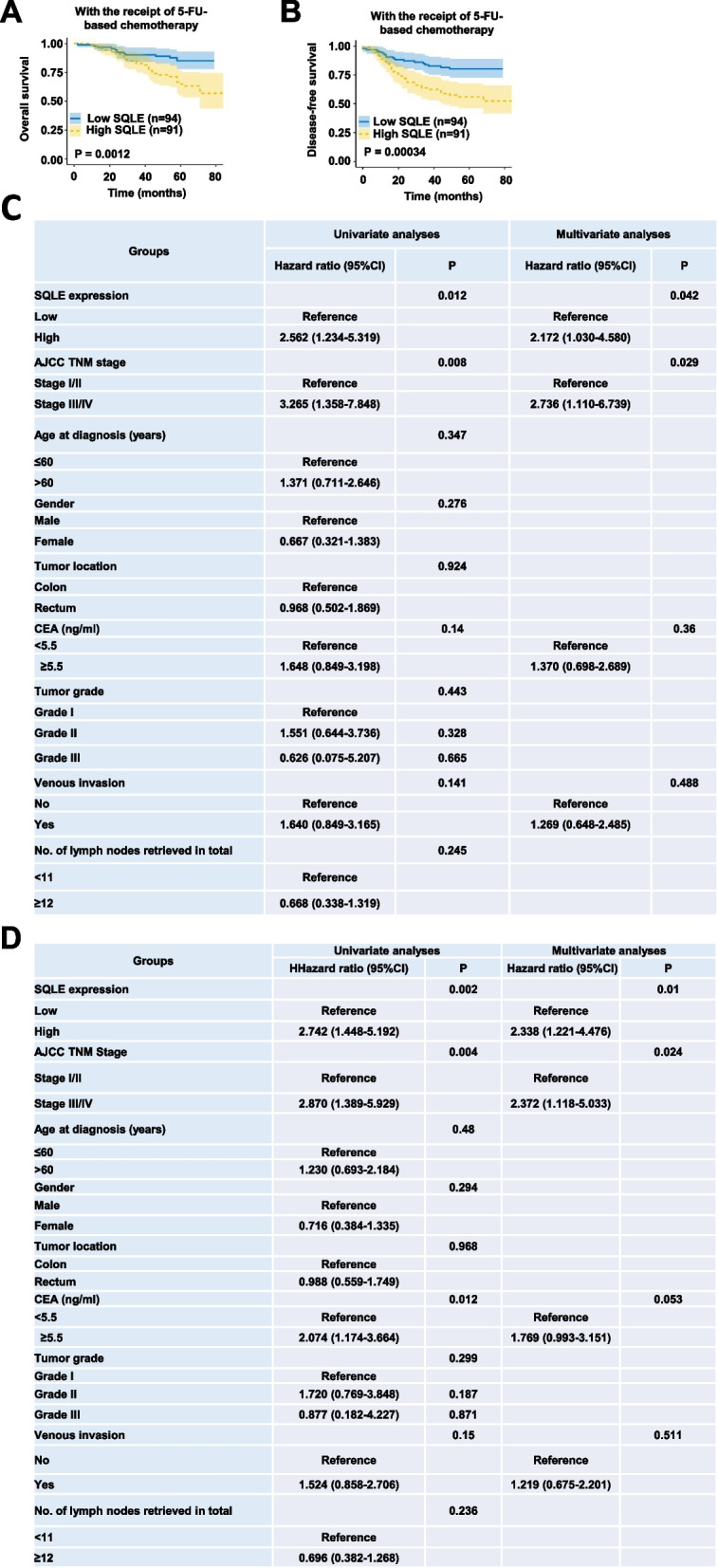
SQLE expression correlates with the response of CRC patients to 5-FU-based chemotherapy. **A**-**B** IHC analyses of SQLE were performed in specimens of CRC patients from FUSCC. OS (**A**) and DFS (**B**) of 185 CRC patients who have received 5-FU-based chemotherapy with low (n = 94) or high (n = 91) expression of SQLE were compared (Kaplan–Meier method with log-rank tests). OS, overall survival. DFS, disease-free survival. **C**-**D** Univariate and multivariate Cox regression analyses (with HR and 95% CI) of OS (**C**) or DFS (D) in 159 CRC patients with the receipt of 5-FU-based chemotherapy. (*p < 0.05; ** p < 0.01)

We next selected 185 CRC patients who had received 5-FU-based chemotherapy from this cohort (Fig. S1F). Kaplan–Meier survival analysis showed that the OS (5-year OS rate: 65.0% vs. 85.1%, *P* = 0.0012) and DFS (5-year DFS rate: 55.9% vs.80.3%, *P* = 0.00034) of CRC patients with high SQLE expression were significantly worse than those with low SQLE expression (Fig. 1A-B). Then we excluded the cases without tumor grade, vascular invasion or preoperative CEA information, and eventually identified 159 CRC patients. The analysis showed that there was no significant difference in tumor stage, age, gender, tumor location, preoperative CEA level, tumor grade, venous invasion and the number of lymph nodes retrieved in total between high SQLE expression and low SQLE expression groups (Fig. S1G). However, there was a significant difference in mortality status between the two groups: the probability of death of patients with high expression of SQLE at the end of the follow-up period was much higher than that of patients with low expression of SQLE (30.6% vs.13.5%, *P* = 0.010). Only the clinicopathological factors with P value less than 0.2 in univariate Cox proportional hazard analysis were then included in the multivariate Cox proportional hazard analysis, which also showed that the OS (HR = 2.172, 95%CI = 1.030–4.580, Fig. 1C) and DFS (HR = 2.338, 95%CI = 1.221–4.476, Fig. 1D) of CRC patients with high expression of SQLE were significantly worse than that of those CRC patients with low expression of SQLE. Collectively, these results suggest the significance of SQLE in 5-FU resistance among CRC patients by indicating that those with elevated SQLE expression experience poorer prognosis with the treatment of 5-FU-based chemotherapy, which is overlooked in previous studies.

### Knockdown of SQLE enhances the sensitivity of CRC to 5-FU treatment

**Fig. 2:**
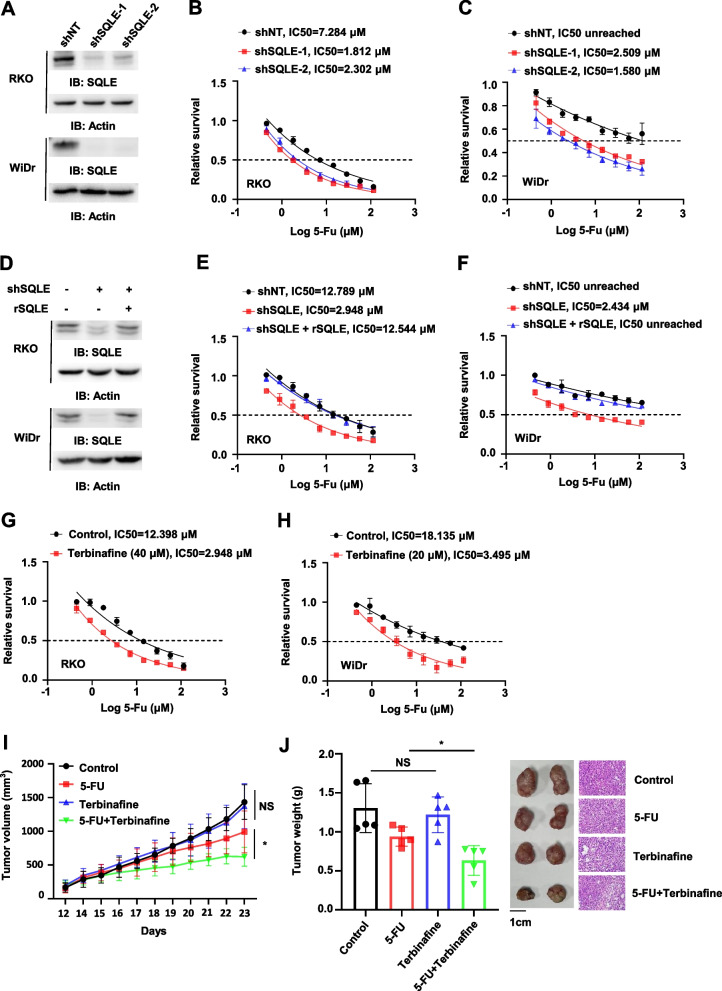
Knockdown of SQLE enhances the sensitivity of CRC to 5-FU treatment. **A** RKO and WiDr cells were infected with the lentivirus expressing non-targeting shRNA (shNT) or shSQLE (including shSQLE-1 and shSQLE-2). Immunoblotting analyses were performed in these cells with indicated antibodies. **B**-**C** The proliferation capacity was tested in RKO (B) and WiDr (C) cells expressing shNT or shSQLE with the treatment of gradient concentration of 5-FU (0–115.2 μM) for 48 h. **D** RKO and WiDr cells were depleted of endogenous SQLE and rescued with Flag-rSQLE. Immunoblotting analyses were performed in these cells. **E**–**F** The proliferation capacity was tested in RKO (E) and WiDr (F) cells with knockdown of endogenous SQLE and rescued expression of Flag-rSQLE with the treatment of gradient concentration of 5-FU (0–115.2 μM) for 48 h. **G**-**H** The proliferation capacity was tested in RKO (G) and WiDr (H) cells with or without the treatment of terbinafine and with the treatment of gradient concentration of 5-FU (0–115.2 μM) for 48 h. **I**-**J** RKO cells were subcutaneously injected into randomized athymic nude mice (5 mice per group) with or without the treatment of terbinafine (100 mg, once/day, 10 days) and 5-FU (40 mg, twice/week, 2 weeks). Tumor growth was measured overtime (I). 23 days after the injection, tumors were dissected for weight measurement and hematoxylin-eosin (HE) staining. Quantitative analyses of dissected tumor weight were performed (left panel), and representative images of HE staining of dissected tumors were shown (right panel) (J). Data represented the means ± SD of 5 mice (unpaired Student’s t test or Mann–Whitney test, two-tailed). (NS, not significant; * p < 0.05)

To confirm the role of SQLE in 5-FU resistance of CRC cells, we depleted SQLE in RKO or WiDr CRC cells by using two small hairpin RNAs (shRNA), including shSQLE-1 and shSQLE-2 (Fig. 2A). Sulforhodamine B (SRB) assays indicated that knockdown of SQLE increased the sensitivity of tumor cells to 5-FU treatment (Fig. 2B-C). In addition, we rescued SQLE-depleted RKO or WiDr cells with shRNA-resistant (r)-SQLE (Fig. 2D) and determined 5-FU sensitivity of these cells. SRB assays indicated that rescued expression of rSQLE recovered the resistance of SQLE-depleted tumor cells to 5-FU, excluding the possibility of off-target effect of SQLE shRNA (Fig. 2E-F).

We next treated RKO and WiDr cells with or without SQLE inhibitor, terbinafine. The inhibition of SQLE increased the sensitivity of RKO and WiDr cells to 5-FU treatment (Fig. 2G-H). Similar results were obtained in the experiments using another inhibitor of SQLE, NB-598, showing that NB-598 treatment could also increase the sensitivity of RKO and WiDr cells to 5-FU treatment (Fig. S2A-B).

Given that the significant toxic and side effects of NB-598 were observed in animal experiments, terbinafine was then used in the subsequent animal experiments [26]. RKO cells were subcutaneously implanted into randomized athymic nude mice. The mice were then treated with or without 5-FU in the absence or presence of terbinafine. Tumor growth was measured by caliper measurements. As shown in Fig. 2I-J, terbinafine treatment greatly improved the antitumoral efficacy of 5-FU treatment. Collectively, these results demonstrate that SQLE expression enhances the resistance of CRC to 5-FU treatment.

### SQLE enhances 5-FU resistance of CRC by upregulating BIRC3 expression

**Fig. 3:**
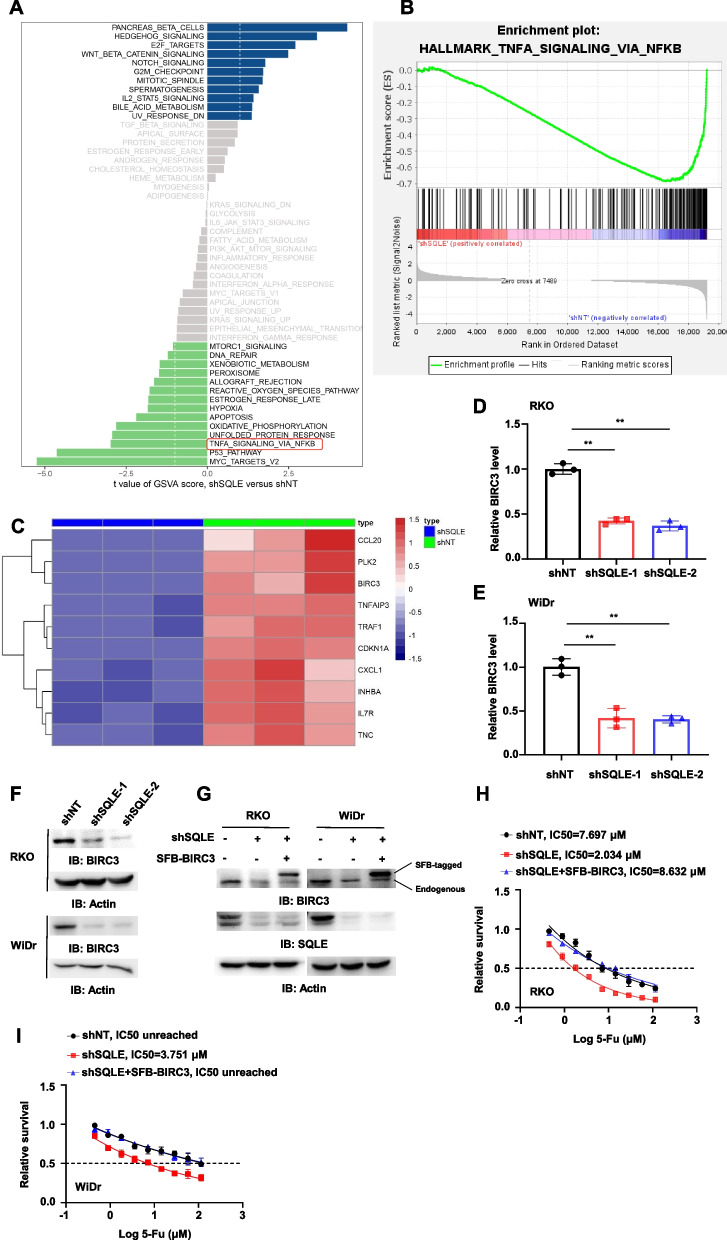
SQLE enhances 5-FU resistance of CRC by upregulating BIRC3 expression. **A** RNA sequencing was conducted on RKO cells with or without SQLE knockdown. Differences in pathway activities were scored by GSVA between shSQLE and shNT cells (NO. of sample = 3 VS. 3). Shown are t values from a linear model (dn, down; UV, ultraviolet; v1, version 1; v2, version 2). **B** GSEA was used to analyze the enriched signaling pathways in NF-κB pathway between shSQLE and shNT groups. NES (normalized enrichment score) = -2.32, FDR (false discovery rate) q-value = 0.0. **C** The heat map shows the top 10 genes which contributed most to the downregulation of NF-κB pathway following SQLE knockdown. Blue and red represent low and high levels, respectively. **D**-**E** Real-time PCR analyses were performed in RKO (D) and WiDr (E) cells with or without SQLE knockdown, and relative BIRC3 mRNA levels was shown. β-actin was used to normalize samples. **F** BIRC3 expression was examined using immunoblotting analyses in RKO and WiDr cells with or without SQLE knockdown. **G**-**I** SQLE-depleted RKO and WiDr cells were infected with the lentivirus expressing EV (empty vector) or SFB-BIRC3. Immunoblotting analyses were performed in these cells with indicated antibodies (G). The proliferation capacity was tested in RKO (H) and WiDr (I) cells with knockdown of endogenous SQLE and overexpression of SFB-BIRC3 with the treatment of gradient concentration of 5-FU (0–115.2 μM) for 48 h

To explore the mechanism of SQLE-promoted 5-FU resistance of CRC, we performed RNA-sequencing analysis in RKO cells with or without SQLE depletion. SQLE depletion resulted in up-regulation of 148 genes and down-regulation of 384 genes in tumor cells after 5-FU treatment (Fig. S3A). Heat map analysis of differential expressions of the genes was shown in Fig. S3B. Furthermore, gene set variation analysis (GSVA) showed that among the signal pathways, NF-κB pathway was the one mostly related to cell survival (Fig. 3A). Gene set enrichment analysis (GSEA), showed that depletion of SQLE significantly down-regulated NF-κB pathway (Fig. 3B). We selected top 10 genes in down-regulated NF-κB pathway after SQLE depletion. Among the genes, BIRC3 was the well-known downstream gene of NF-κB pathway related to 5-FU resistance [27, 28] (Fig. 3C). Quantitative PCR (qRCR) analyses and immunoblotting analyses showed that knockdown of SQLE dramatically decreased the mRNA and protein levels of BIRC3 (Fig. 3D-F). We then overexpressed S-FLAG-streptavidin-binding peptide (SFB)-tagged BIRC3 in SQLE-depleted RKO and WiDr cells (Fig. 3G). BIRC3 overexpression completely abrogated SQLE depletion-enhanced sensitivity of CRC cells to 5-FU treatment (Fig. 3H-I), suggesting that SQLE promoted the resistance of CRC to 5-FU treatment by upregulating BIRC3 expression.

### SQLE activates NF-κB pathway to upregulate BIRC3 and enhance 5-FU resistance of CRC

**Fig. 4:**
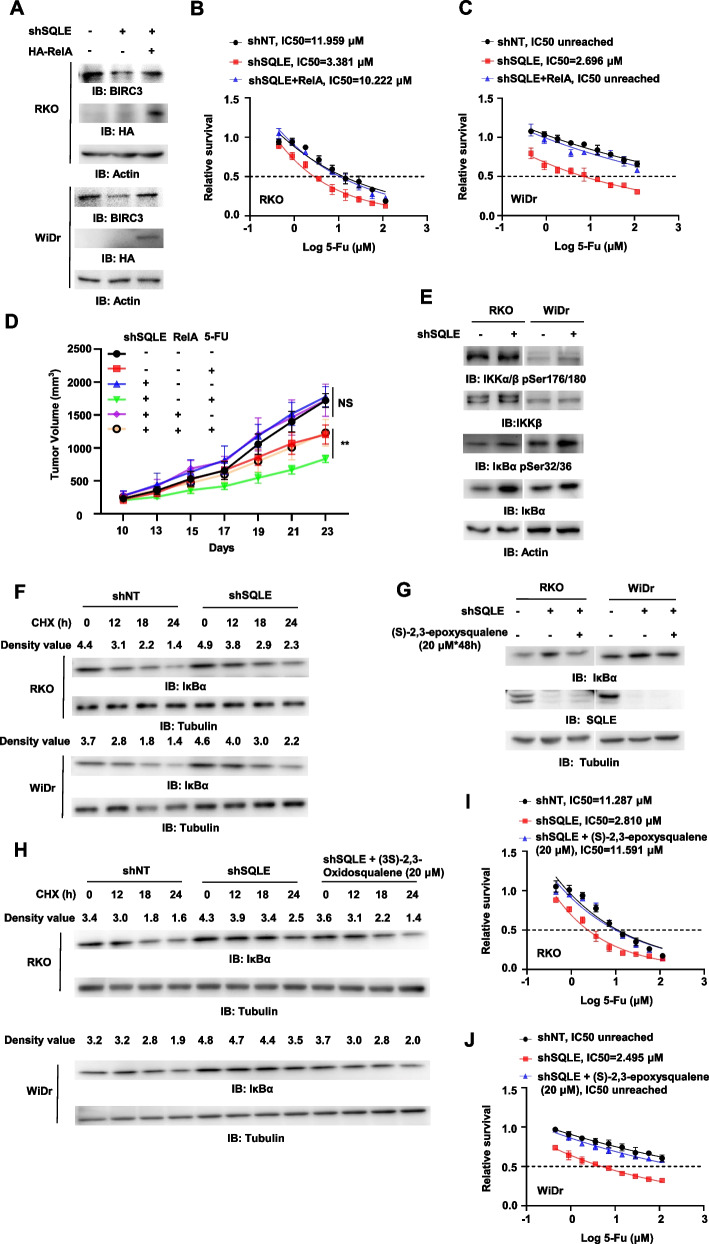
SQLE activates NF-κB pathway to upregulate BIRC3 and enhance 5-FU resistance of CRC. **A**-**C** RKO and WiDr cells were depleted of endogenous SQLE and overexpressed with HA-RelA. Immunoblotting analyses were performed in these cells with indicated antibodies (**A**). The proliferation capacity was tested in RKO (**B**) and WiDr (**C**) cells with knockdown of endogenous SQLE and overexpression of HA-RelA with the treatment of gradient concentration of 5-FU (0–115.2 μM) for 48 h. **D** RKO cells were depleted of endogenous SQLE and overexpressed with HA-RelA. These cells were subcutaneously injected into randomized athymic nude mice (5 mice per group) with or without the treatment of 5-FU (40 mg, twice/week, 2 weeks). Tumor growth was measured by caliper measurements (unpaired Student’s t test or Mann–Whitney test, two-tailed). (NS, not significant; ** p < 0.01). **E** Immunoblotting analyses were performed in RKO and WiDr cells with or without SQLE knockdown. **F** Immunoblotting analyses were performed in RKO and WiDr cells with or without SQLE depletion and treated with cycloheximide (CHX; 25 μg/mL) for 0, 12, 18, and 24 h. **G** SQLE-depleted RKO and WiDr cells were treated with or without (S)-2,3-epoxysqualene (20 μM) for 48 h. Immunoblotting analyses were performed in these cells with indicated antibodies. **H** SQLE-depleted RKO and WiDr cells were treated with (S)-2,3-epoxysqualene (20 μM) for 48 h. Immunoblotting analyses were performed in these cells treated with CHX (25 ug/mL) for 0, 12, 18, and 24 h. **I-J** SQLE-depleted RKO (I) and WiDr (J) cells were treated with (S)-2,3-epoxysqualene (20 μM) for 72 h. The proliferation capacity was tested in these cells with the treatment of gradient concentration of 5-FU (0–115.2 μM) for 48 h

To test whether SQLE upregulates BIRC3 expression through NF-κB, we overexpressed HA-RelA, the central transcription factor in NF-κB pathway, in SQLE-depleted tumor cells. RelA overexpression recovered BIRC3 expression in SQLE-depleted RKO and WiDr cells (Fig. 4A). Moreover, RelA overexpression restored the resistance of SQLE-depleted CRC cells to 5-FU treatment (Fig. 4B-C).

In addition, we subcutaneously implanted SQLE-depleted RKO cells with or without HA-RelA overexpression into randomized athymic nude mice. The mice were treated with or without 5-FU and tumor growth was measured by caliper measurements. RelA overexpression completely restored the resistance of SQLE-depleted CRC cells to 5-FU treatment (Fig. 4D and Fig. S4A).

Collectively, these results suggest that SQLE promotes the resistance of CRC to 5-FU treatment by activating NF-κB signaling.

### (S)-2,3-epoxysqualene activates NF-κB pathway by accelerating IκBα degradation

To investigate how SQLE activates NF-κB pathway, immunoblotting analyses were performed to examine the activation of NF-κB in RKO and WiDr cells. As shown in Fig. 4E, SQLE depletion significantly increased IκBα expression, without influencing the level of phospho-IKKα/β (Ser176/180) and IKKβ, suggesting that SQLE depletion-inactivated NF-κB pathway was not through IKKα/β. Phosphorylation of IκBα leads to its ubiquitination and proteasomal degradation to liberate NF-κB and allows NF-κB translocation to the nucleus to control the expression of the target genes [29]. We speculated that SQLE activates NF-κB by regulating ubiquitination-mediated IκBα degradation. To test the hypothesis, we treated RKO or WiDr cells with or without SQLE depletion with CHX, (25 μg/mL). SQLE depletion significantly slowed down the degradation of IκBα (Fig. 4F). In addition, SQLE inhibition with NB-598 also enhanced protein stability of IκBα in RKO and WiDr cells (Fig. S4B).

SQLE is an important metabolic enzyme in the mevalonate pathway of cholesterol synthesis, and (S)-2,3-epoxysqualene is the direct product of squalene catalyzed by SQLE. To investigate the mechanism of SQLE-regulated IκBα degradation, we examined whether (S)-2,3-epoxysqualene regulated IκBα degradation. The addition of (S)-2,3-epoxysqualene (20 μM, 48 h) decreased protein levels and protein stability of IκBα in RKO and WiDr cells (Fig. 4G and H). Consequently, the addition of (S)-2,3-epoxysqualene (20 μM, 48 h) restored the resistance of SQLE-depleted CRC cells to 5-FU treatment (Fig. 4I-J).

Collectively, these results suggest that SQLE promotes 5-FU resistance of CRC cells through (S)-2,3-epoxysqualene-regulated IκBα degradation.

### (S)-2,3-epoxysqualene enhances the interaction between BTRC and IκBα

**Fig. 5:**
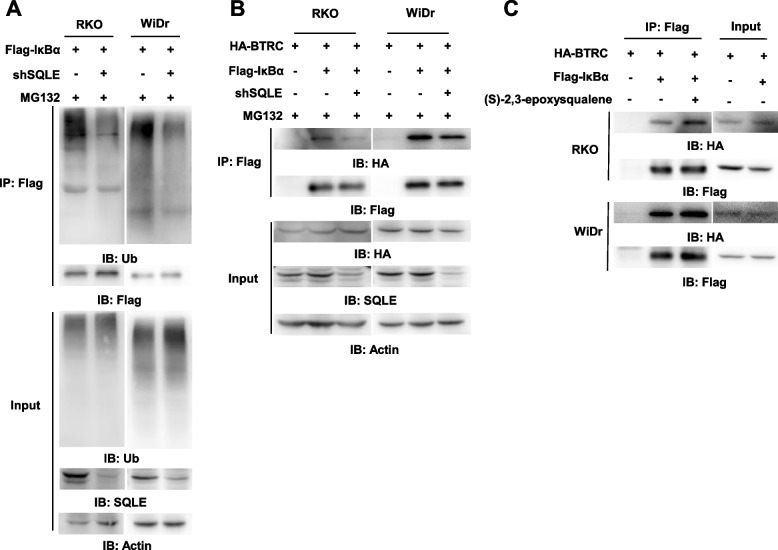
(S)-2,3-epoxysqualene enhances the interaction between BTRC and IκBα. **A** RKO and WiDR cells stably expressing Flag-IκBα were infected with the lentivirus expressing shNT or shSQLE. Cells were treated with MG132 (20 μM, 6 h). Flag-IκBα proteins were immunoprecipitated using anti-Flag agarose beads. **B** RKO and WiDR cells stably expressing HA-BTRC were infected with the lentivirus expressing EV or Flag-IκBα and the lentivirus expressing shNT or shSQLE. Cells were treated with MG132 (20 μM, 6 h). Flag-IκBα proteins were immunoprecipitated using anti-Flag agarose beads. **C** RKO and WiDR cells stably expressing HA-BTRC were infected with the lentivirus expressing EV or Flag-IκBα. Cells were harvested and (S)-2,3-epoxysqualene were added to the cell lysates before immunoprecipitation using anti-Flag agarose beads

Since the ubiquitin–proteasome system is the most classical degradation way of IκBα, we further explore the effect of SQLE depletion on the ubiquitin of IκBα by using immunoprecipitation assay. Immunoblotting analyses showed that the ubiquitin level of immunoprecipitated IκBα was much lower in RKO and WiDr cells with SQLE depletion, suggesting that SQLE knockdown slowed down the degradation of IκBα through ubiquitin–proteasome system (Fig. 5A). BTRC was the E3 ubiquitin ligase of IκBα [30], we then tested whether SQLE knockdown influenced the interaction between BTRC and IκBα. As shown in Fig. 5B, SQLE knockdown inhibited the interaction between IκBα and BTRC in both RKO and WiDr cells. Interestingly, (S)-2,3-epoxysqualene treatment enhanced the interaction between IκBα and BTRC in RKO and WiDr cells (Fig. 5C). These results support that SQLE-produced (S)-2,3-epoxysqualene enhances the interaction between IκBα and BTRC to accelerate IκBα degradation.

### SQLE expression correlates with IκBα and BIRC3 expression in CRC

**Fig. 6:**
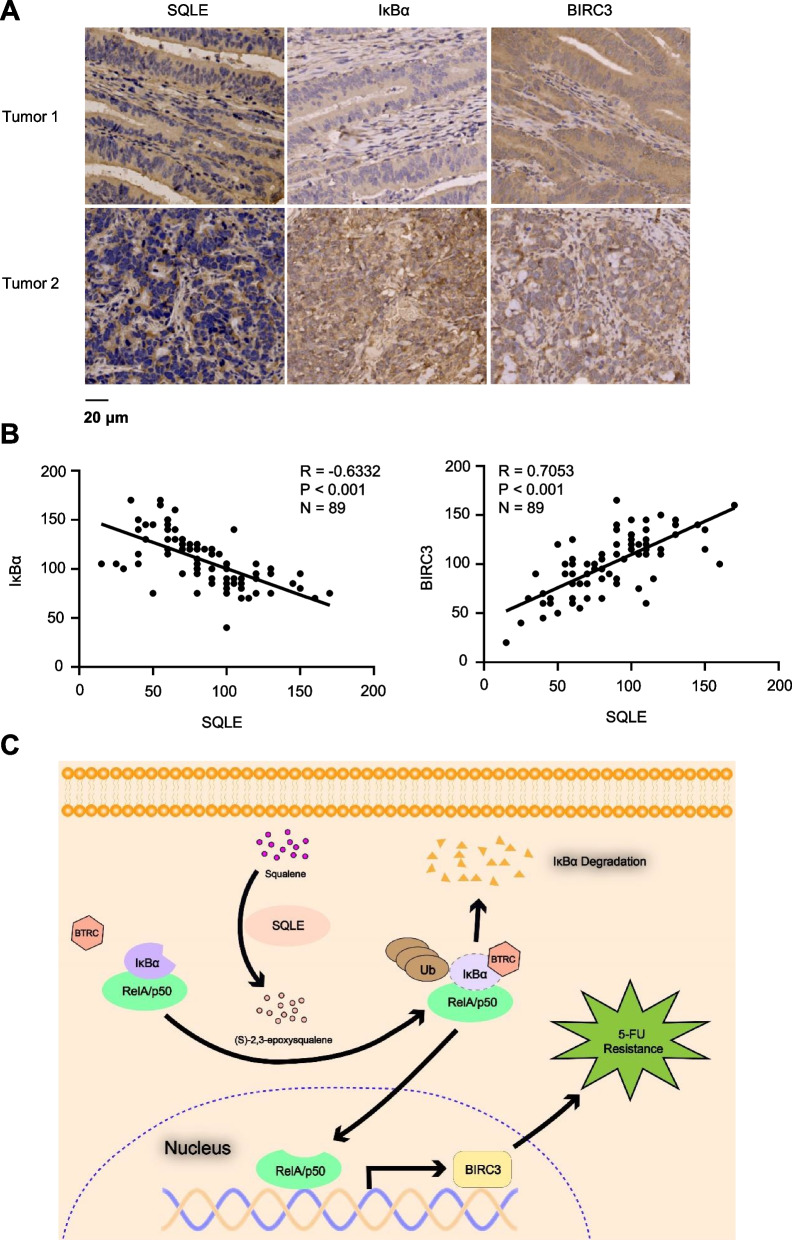
SQLE expression correlates with IκBα and BIRC3 expression in CRC. **A**-**B** IHC analyses using anti-SQLE, anti-IκBα and anti-BIRC3 antibodies were performed in tumor tissues from CRC patients. SQLE mainly localized in the cytosol, while IκBα and BIRC3 localized in both cytosol and nucleus. Representative images of IHC staining were shown (**A**). Semiquantitative scoring (H score) was carried out and correlations between SQLE and IκBα or SQLE and BIRC3 expression were analyzed (Pearson product moment correlation test, **B**). **C** A schematic model of SQLE-promoted resistance of CRC to 5-FU by (S)-2,3-epoxysqualene-activated NF-κB. SQLE-produced (S)-2,3-epoxysqualene enhances the binding of IκBα to BTRC, which promotes the degradation of IκBα and the activation of NF-κB pathway. Activated NF-κB upregulates BIRC3 expression to promote the resistance of CRC to 5-FU

To define the clinical relevance of SQLE-regulated NF-κB signaling and BIRC3 expression, we performed IHC analysis in tumor tissues from CRC patients with anti-SQLE, anti-IκBα and anti-BIRC3 antibodies. SQLE protein levels exhibited positive correlation with BIRC3 protein levels, but exhibited negative correlation with IκBα levels in clinical CRC specimens (Fig. 6A-B).

## Discussion

Some previous studies have shown that cholesterol contributes to chemotherapy resistance, thereby carrying prognostic impact for cancer patients [17, 31]. However, apart from cholesterol itself, there is a lack of evidence regarding the relationship between metabolic intermediates of cholesterol metabolism and chemotherapy. For the first time, in this study, we found and proved the inhibitory role of SQLE in the drug sensitivity of CRC to 5-FU, highlighting SQLE as a potential target for CRC treatment. Further investigation into the underlying mechanism revealed that the direct product of SQLE-catalyzed squalene, (S)-2,3-epoxysqualene, could enhance the binding of IκBα to BTRC, leading to the upregulation of ubiquitin levels and subsequent degradation of IκBα. This activation of the NF-κB pathway ultimately inhibits the sensitivity of CRC to 5-FU (Fig. 6C). Apart from providing a new potential therapeutic target for colorectal cancer, more importantly, our study has established the first association between a metabolic intermediate of cholesterol metabolism and the NF-κB pathway.

5-FU, an early-reported chemotherapeutic drug, remains a cornerstone in the systemic treatment of CRC. Despite advancements in cancer treatment, the prognosis of CRC patients receiving 5-FU therapy remains unsatisfactory [32]. Therefore, it is crucial to explore and enhance the sensitivity of 5-FU to better design appropriate treatment strategies and achieve personalized care that effectively improves patient outcomes. SQLE is the second key enzyme in cholesterol biosynthesis and catalyzes the stereospecific oxidation of squalene to (S)-2,3-epoxysqualene. The correlation between the high expression of SQLE and the poor prognosis of patients with malignant tumor has been reported for a long time [13, 23, 24, 33–35]. In CRC, low mRNA level of SQLE is associated with a better prognosis of patients [23, 36]. CRC patients with high expression of SQLE have shorter recurrence-free survival (RFS) and overall survival (OS), highlighting SQLE as a prognostic biomarker for CRC [35]. It is suggested that the poor prognosis of patients with high expression of SQLE is attributed to its promotion of tumor cell proliferation [24, 33]. Recently, in CRC, it has been reported that inhibition of SQLE could reduce the levels of calcitriol (the active form of vitamin D3) and CYP24A1, and then increase the intracellular Ca^2+^ concentration, thus inhibiting the MAPK signal pathway, resulting in the suppression of CRC cell growth [24]. However, no previous studies investigated the effect of SQLE on CRC treatment efficacy. For the first time, therefore, we found and demonstrated that SQLE could inhibit the drug sensitivity of CRC to 5-FU, contributing to the poor prognosis in CRC patients, which also provided new evidence for the correlation between high expression of SQLE and adverse patient outcomes.

(S)-2,3-epoxysqualene is the direct product of squalene catalyzed by SQLE in cholesterol metabolism, however, little information is currently available about the value of (S)-2,3-epoxysqualene in cancer research. Only one previous study has reported that (S)-2,3-epoxysqualene would arrest the cell cycle in the G0/G1 phase and induce cell apoptosis in glioblastoma multiforme [22]. In our study, we experimentally demonstrated that (S)-2,3-epoxysqualene treatment would result in the activation of NF-κB pathway. Therefore, we establish for the first time a direct relationship between a small molecule of cholesterol metabolism and NF-κB pathway that (S)-2,3-epoxysqualene could enhance the binding of IκBα to BTRC to up-regulate the ubiquitin level of IκBα, thus promoting the degradation of IκBα, which leads to the activation of NF-κB pathway and the up-regulation of BIRC3, and ultimately inhibits the drug sensitivity of 5-FU. Our results have uncovered a unique molecular function of (S)-2,3-epoxysqualene to activate NF-κB pathway and the critical role of (S)-2,3-epoxysqualene-activated NF-κB in 5-Fu treatment of CRC.

As early as 2009, some researchers found that the expression of BIRC3 in CRC tissues was higher than that in normal tissues [37]. More importantly, cancer patients with high expression of BIRC3 exhibited an increased risk of early recurrence following fluorouracil treatment. Down-regulation of BIRC3 could activate caspase3/7, promote apoptosis and effectively increase the sensitivity of CRC cells to 5-FU [37]. Down-regulation of BIRC3 can enhance the drug sensitivity of 5-FU in oral squamous cell carcinoma, esophageal adenocarcinoma and other malignant tumors in later studies [38, 39]. In the present study, our results are in agreement with these studies, which again emphasizes the important role of BIRC3 in drug sensitivity.

Terbinafine is an inhibitor of SQLE, which plays a non-competitive inhibitory effect on SQLE by inhibiting squalene entering the active site of SQLE, thus inhibiting the synthesis of ergosterol and playing a bactericidal effect [40]. Although human cells are less sensitive to terbinafine than fungi, it demonstrates good tolerance and fewer adverse reactions in clinical applications for fungal infections, making it a promising candidate for clinical application in oncology [21]. Previous studies have reported that terbinafine could inhibit the proliferation of tumor cells and have potential anti-tumor effect [24, 33]. Another retrospective study covering the Swedish population showed that prostate cancer patients treated with oral terbinafine had a 47% lower risk of mortality from prostate cancer and a 36% lower risk overall mortality than the control group [41]. A recent study found that drug inhibition of SQLE leaded to squalene accumulation and subsequent endoplasmic reticulum stress, resulting in increased radiosensitivity of breast and lung cancer cells [42]. In addition, Li et al. [43] reported that terbinafine in combination with 5- FU or oxaliplatin could synergistically suppress colorectal cancer growth by regulating gut barrier function, which also supported our findings in the present study.

In summary, our discovery unveils a novel mechanism that (S)-2,3-epoxysqualene-dependent NF-κB activation promotes 5-FU resistance of CRC, which establishes the first association between a metabolic intermediate of cholesterol metabolism and the NF-κB pathway. Moerover, the various potential anti-tumor effects of terbinafine suggest its high potential as an anticancer drug, generating significant interest in its clinical application in CRC patients, and related clinical work is also being actively carried out.

### Supplementary Information

Supplementary Material 1.

## Data Availability

The datasets used and analyzed during the current study are available from the corresponding author on reasonable request. The RNA-sequencing data reported in this paper are publicly available in the National Center for Biotechnology Information’s Gene Expression Omnibus (GEO) database. The accession code for the RNA-sequencing data is GEO GSE232678.
