# Supplementary material for: Squalene epoxidase promotes the chemoresistance of colorectal cancer via (S)-2,3-epoxysqualene-activated NF-κB
Source: Cell Commun Signal. 2024 May 18;22:278. doi: 10.1186/s12964-024-01649-z (PMC11102232; doi:10.1186/s12964-024-01649-z)
Supplement: Supplementary file 1 — Supplementary Material 1. [file 12964_2024_1649_MOESM1_ESM.docx]

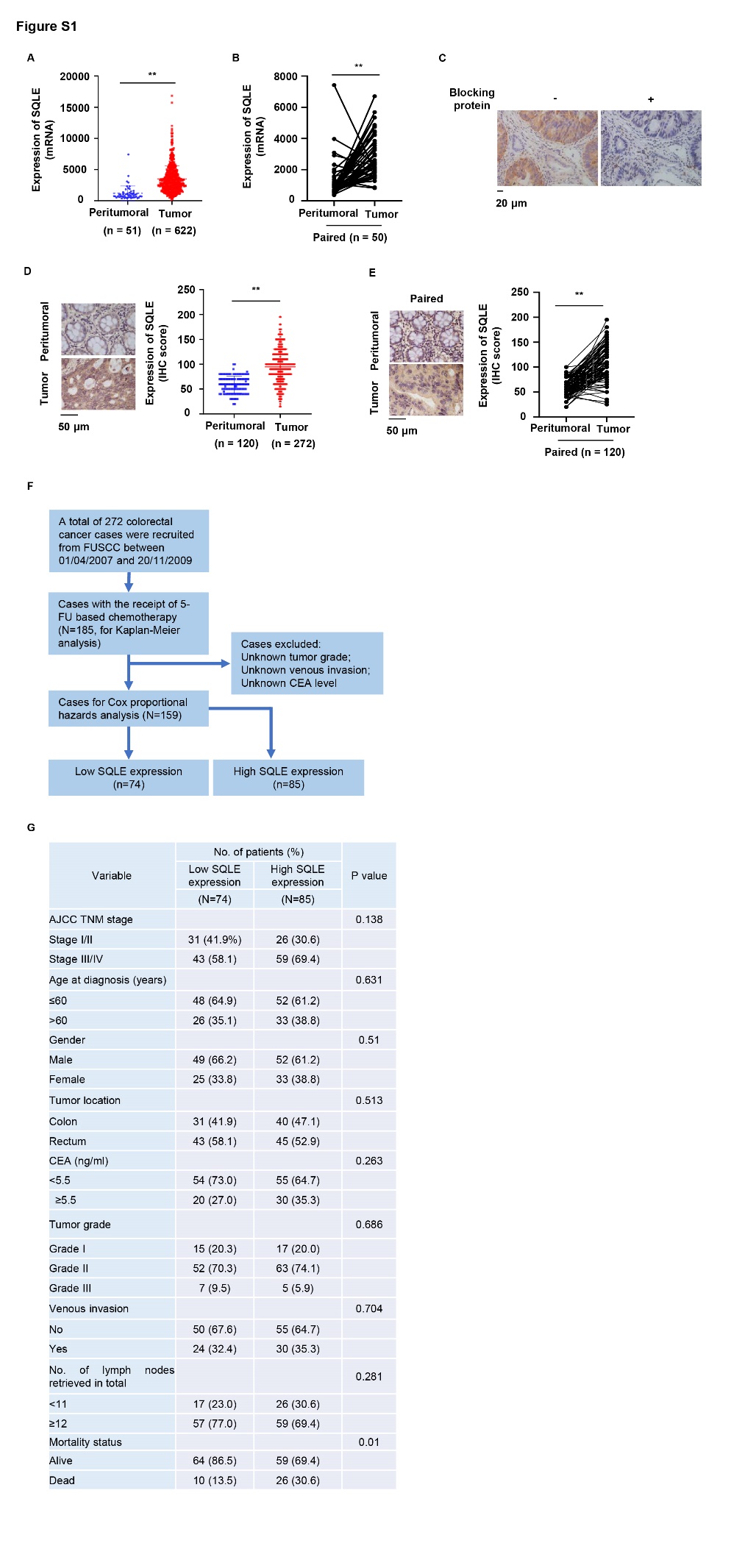


**Figure S1. SQLE expression correlates with the response of CRC patients to 5-FU-based chemotherapy. Related to Figure 1.**

(A–B) TCGA RNA-sequencing data of patients with CRC were analyzed. *SQLE* mRNA levels were compared between tumors and peritumoral tissues (Mann-Whitney test, two-tailed, A) or between tumors and paired peritumoral tissues (Wilcoxon signed-rank test, two-tailed, B). Data represented means ± SD.

(C) IHC analyses of CRC tissues using anti-SQLE antibody were performed with or without SQLE blocking protein.

(D-E) IHC analyses of SQLE were performed in specimens of CRC patients from FUSCC. The expression of SQLE between tumors and peritumoral tissues (Mann-Whitney test, two-tailed, D) or between tumors and paired peritumoral tissues (Wilcoxon signed-rank test, two-tailed, E) were compared. Data represented means ± SD. Left, representative IHC staining images. Right, semiquantitative scoring (H score) was performed.

(F) Flow chart of the CRC patients selection process in FUSCC.

(G) Comparison of different clinicopathological characteristics between low (n=74) or high (n=85) expression of SQLE groups in 159 CRC patients with the receipt of 5-FU-based chemotherapy (chi-square test).


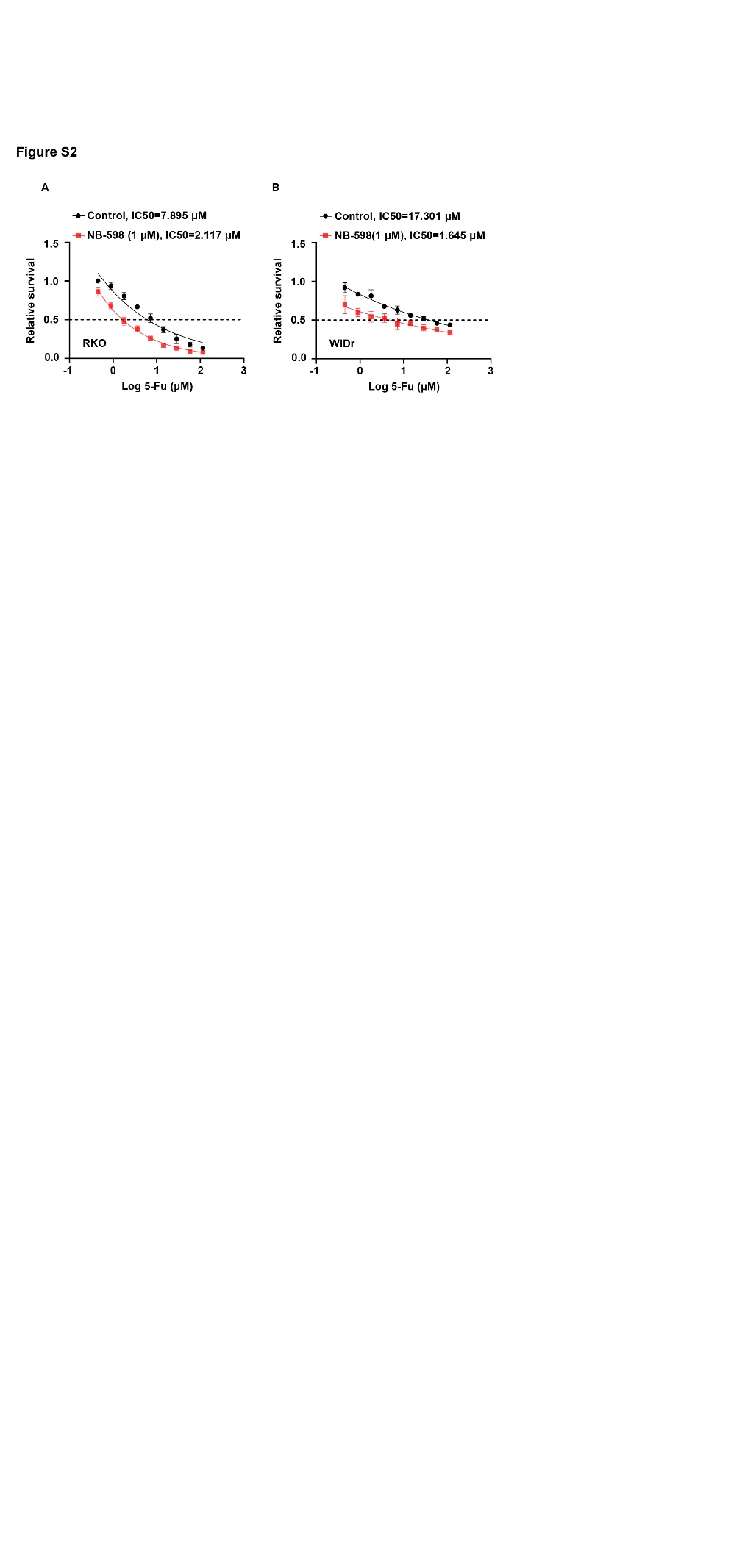


**Figure S2. Inhibiton of SQLE enhances the sensitivity of CRC to 5-FU treatment. Related to Figure 2.**

(A-B) The proliferation capacity was tested in RKO (A) and WiDr (B) cells with or without the treatment of NB-598 (1 μM, 72 h) and with the treatment of gradient concentration of 5-FU (0-115.2 μM) for 48 hours.


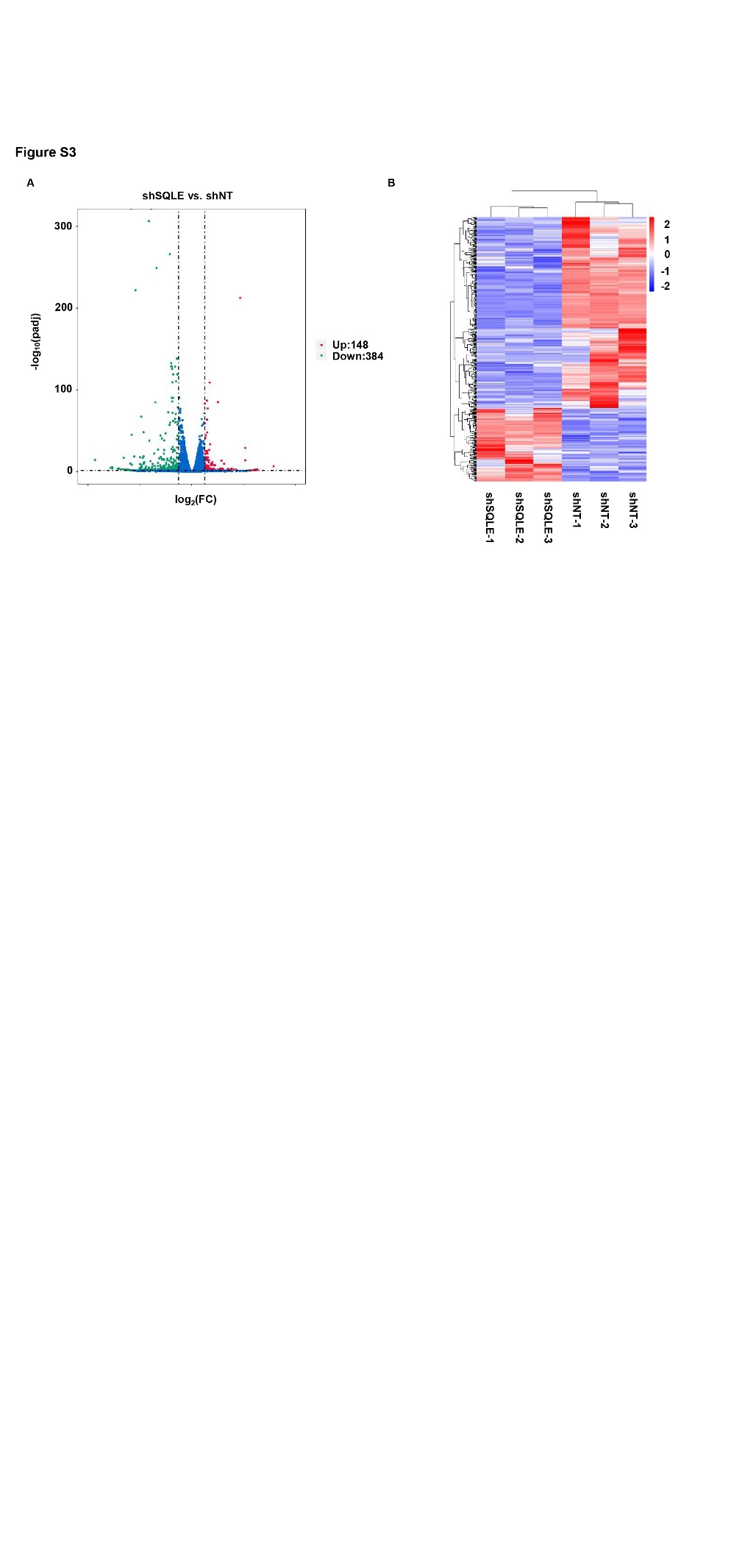


**Figure S3. SQLE enhances 5-FU resistance of CRC by upregulating BIRC3 expression. Related to Figure 3.**

(A) Volcano plot of the RNA-sequencing data analysis. The horizontal axis represented the multiple values of gene expression difference between the two samples (log2FoldChange), and the vertical axis indicated the significant levels of different gene expression (- log10padj). The up-regulated genes were represented by red dots, the down-regulated genes were represented by green dots, and the genes with no significant changes were represented by blue dots.

(B) Differential gene heat map analysis, also known as differential gene cluster analysis. Heat map color represented the level of gene expression. The horizontal axis represented the clustering results of samples before and after knockdown of SQLE, and the vertical axis represented the clustering results of different genes. In the heat map, red and blue indicated high and low expression of different genes, respectively.


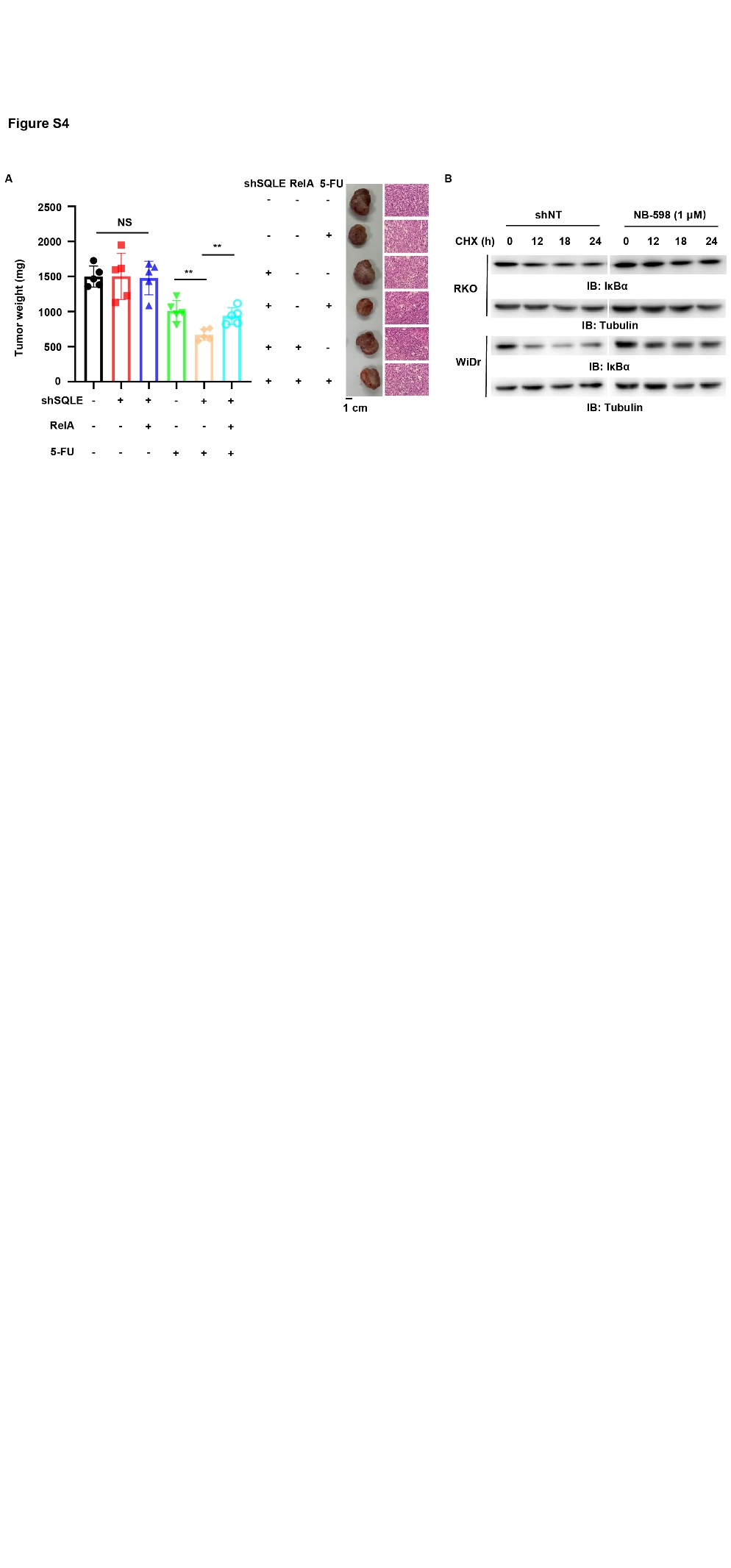


**Figure S4. SQLE activates NF-κB pathway to upregulate BIRC3 and enhance 5-FU resistance of CRC. Related to Figure 4.**

(A) RKO cells were depleted of endogenous SQLE and overexpressed with HA-RelA. These cells were subcutaneously injected into randomized athymic nude mice (5 mice per group) with or without the treatment of 5-FU (40 mg, twice/week, 2 weeks). 23 days after the injection, 23 days after the injection, tumors were dissected for weight measurement and HE staining. Quantitative analyses of dissected tumor weight were performed (left panel), and representative images of HE staining of dissected tumors were shown (right panel). Data represented the means ± SD of 5 mice (unpaired Student’s t test, two-tailed). (NS, not significant; **p < 0.01).

(B) Immunoblotting analyses were performed in RKO and WiDr cells treated with or without NB-598 for 48 h and treated with CHX (25 μg/mL) for 0, 12, 18, and 24 h.

**SUPPLEMENTAL TABLE**

**List of antibodies.**

| **Antibodies** | **Cat. Number** | **Company** |
| --- | --- | --- |
| anti-Flag | F1804 | Sigma |
| anti-HA | 3724 | CST |
| anti-Tubulin | sc-23948 | Santa Cruz |
| BetaActinMouseMonoclonalAntibody | 66009-1-Ig | Proteintech |
| anti-IKBKB | 15649-1-AP | Proteintech |
| anti-Ubiquitin | ab134953 | Abcam |
| anti-SQLE | 12544-1-AP | Proteintech |
| anti-c-IAP2 | #3130 | Cell Signaling Technology |
| anti-phospho-IκBα | #9246 | Cell Signaling Technology |
| anti- IκBα | 10268-1-AP | Proteintech |
| IKKα/β pSer176/180 | 2697S | Cell Signaling Technology |
